# Regulation of TGF-β Superfamily Signaling by SMAD Mono-Ubiquitination

**DOI:** 10.3390/cells3040981

**Published:** 2014-10-15

**Authors:** Feng Xie, Zhengkui Zhang, Hans van Dam, Long Zhang, Fangfang Zhou

**Affiliations:** 1 Life Sciences Institute, Zhejiang University, Hangzhou, Zhejiang 310058, China; 2 Department of Molecular Cell Biology, Cancer Genomics Centre Netherlands and Centre for Biomedical Genetics, Leiden University Medical Center, Postbus 9600 2300 RC Leiden, The Netherlands

**Keywords:** TGF-β, BMP, SMAD, Mono-ubiquitination

## Abstract

TGF-β(transforming growth factor-β) superfamily signaling mediators are important regulators of diverse physiological and pathological events. TGF-β signals are transduced by transmembrane type I and type II serine/threonine kinase receptors and their downstream effectors, the SMAD (drosophila mothers against decapentaplegic protein) proteins. Numerous studies have already demonstrated crucial regulatory roles for modification of TGF-β pathway components by poly-ubiquitination. Recently, several studies also uncovered mono-ubiquitination of SMADs as a mechanism for SMAD activation or inactivation. Mono-ubiquitination and subsequent deubiquitination of SMAD proteins accordingly play important roles in the control of TGF-β superfamily signaling. This review highlights the major pathways regulated by SMAD mono-ubiquitination.

## 1. Introduction

The SMAD proteins are part of the signaling cascades that represent the canonical downstream pathways of transforming growth factor β (TGF-β) super family proteins, which include TGF-βs, Activins, Inhibins, BMPs (bone morphogenetic proteins), GDFs (growth and differentiation factors), and Nodal (nodal growth differentiation factor). Ligand stimulation of type I and type II receptor serine/threonine kinases results in association and activation of receptor complexes in the cell membrane, which, in turn, phosphorylate and thereby activate the receptor regulated SMADs (R-SMADs). R-SMADs form complexes with SMAD4 (a common-partner (co-)SMAD) and translocate to the nucleus where their target genes are activated or inactivated. R- and Co-Smads contain two conserved domains at their amino-terminal and carboxyl-terminal ends, the Mad Homology (MH)1 and MH2 domains, respectively, which are connected by a less conserved linker region. Both the MH1 and MH2 domains mediate interactions of SMADs with other transcription factors, co-activators, co-repressors and chromatin-remodeling factors. The ability of SMADs to interact with other DNA binding factors greatly facilitates gene-regulation, as SMADs bind DNA only with low affinity via MH1. The MH2 domain of R‑Smads also mediates the interaction of R-Smads and type I TGF-β-related receptors prior to phosphorylation [1,2]. 

TGF-β/SMAD signaling plays crucial roles in both embryogenesis and carcinogenesis. In embryonic development, Nodal proteins are essential for mesoderm induction in vertebrates [3,4,5,6]; BMP signals are essential for embryonic patterning and early skeletal formation [7]; TGF-β/Activin regulates vascular function and angiogenesis due to their promotion of both EMT (epithelial to mesenchymal transition) and EndoMT (endothelial to mesenchymal transition) [8,9]. In carcinogenesis, TGF-β/SMAD signaling has dual functions. It can inhibit cell cycle progression or induce apoptosis and, thereby, be cytostatic or cytotoxic in premalignant tumor cells. However, in advanced tumors, in which oncogenic mutations have inactivated these tumor-suppressing functions, TGF-β/SMAD signaling can induce or enhance EMT, invasion, and metastasis [10,11]. 

In this review we will summarize the current knowledge on the role of mono-ubiquitination in the canonical TGF-β/SMAD signaling pathways. For information on other types of regulation and receptor-initiated Non-SMAD signaling, we refer to two recent review articles [12,13]. 

### 1.1. Ubiquitin and Ubiquitination

**Figure 1:**
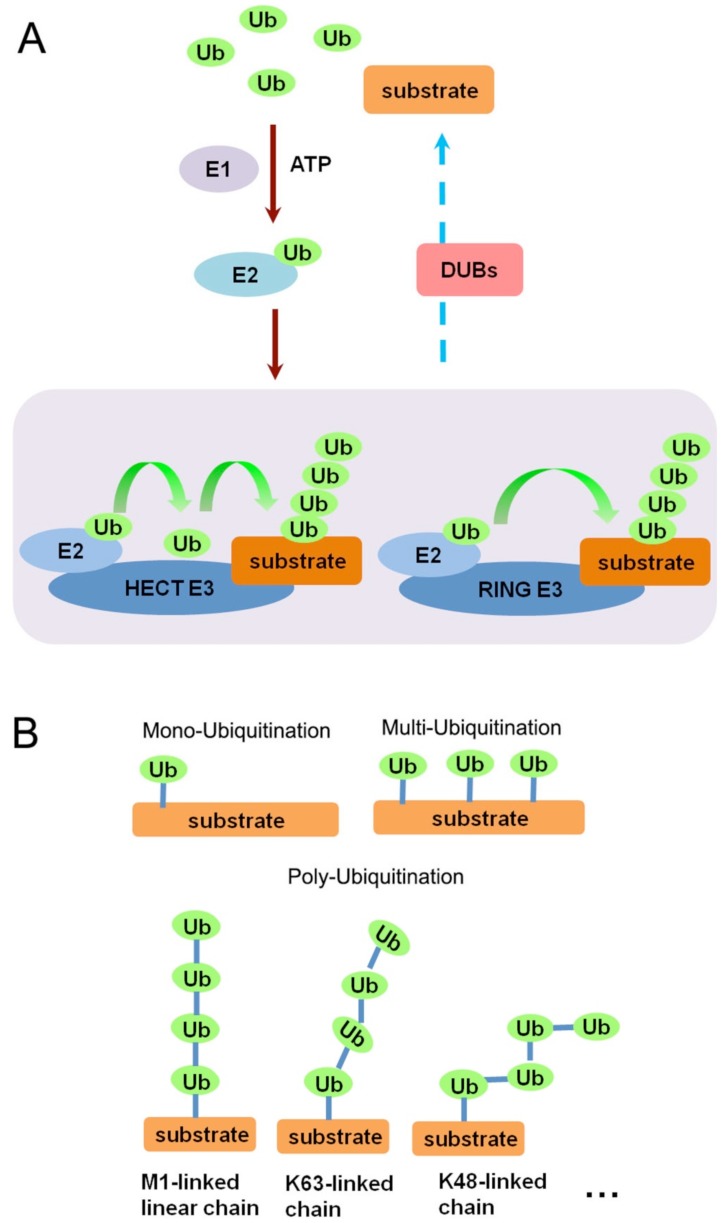
(**A**) Schematic representation of the ubiquitination procedure by E1, E2, and E3 enzymes; (**B**) Schematic representation of different ubiquitination patterns, including mono-ubiquitination, multi-ubiquitination, and poly-ubiquitination.

As a small regulatory protein (76 amino acids), ubiquitin (Ub) exists in almost all kinds of eukaryotic cells. Originally being characterized as a covalently attached signal for proteasomal degradation [14], it has currently been implicated in a wide scope of cellular events, including signaling transduction, protein trafficking, cell cycle regulation, and DNA damage and repair [15,16] Each of the seven lysine residues of ubiquitin can be covalently attached to the carboxyl end of another ubiquitin, enabling different patterns of ubiquitin linkage forms [17]. This linkage (ubiquitination) is an enzymatic post-translational modification process requiring a ubiquitin-activating enzyme (E1), a ubiquitin-conjugating enzyme (E2), and a substrate-specific ubiquitin ligase (E3). As shown in Figure 1A, in the presence of ATP, the E1 enzyme activates the Ub molecule and transfers it to the E2. The substrate specific E3 enzymes (such as E6-AP Carboxyl Terminus (HECT) domain containing E3 ubiquitin ligases or really interesting new gene (RING) domain containing E3 ubiquitin ligases) subsequently facilitate or enable transfer of the ubiquitin from the E2 enzyme to the substrate. In the case of HECT- and RBR (RING-between-RING)-ligases this involves formation of a Ub-E3 thioester intermediate [18,19,20]. Protein substrates can be ubiquitinated in diverse ways, including mono-, multi-, poly-, linear-, and their combinations (Figure 1B). Different linkages of ubiquitin dictate distinct functions. Mono- and multi-ubiquitination can alter protein interactions and localization. Poly-ubiquitins linked through lysine-48 provide the main targeting signals for proteasomal degradation, whereas lysine-63 linkage, as well as linear chains, can make the substrate protein a scaffold in cellular signaling [20]. Importantly, ubiquitination is a reversible process and is counteracted by deubiquitinating enzymes (DUBs), which are proteases that remove ubiquitin from its conjugates and, therefore, perform important regulatory functions in cell signaling and homeostasis. 

### 1.2. TGF-β/SMAD and BMP/SMAD Signaling 

**Figure 2:**
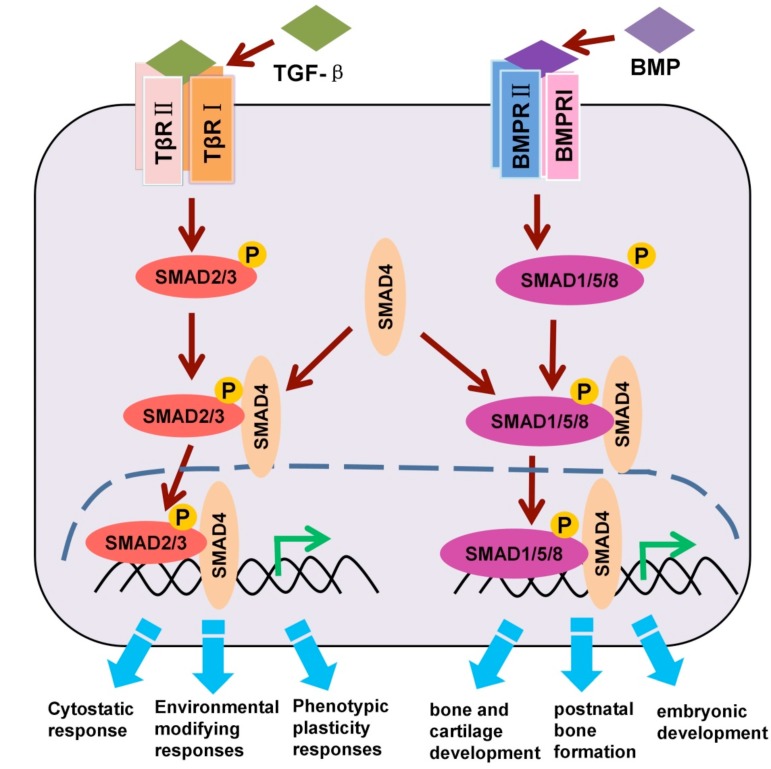
Schematic representation of TGF-β/SMAD and BMP/SMAD activation.

As mentioned in the introduction, TGF-β family ligands bind and activate specific heteromeric type I and type II Ser/Thr kinase receptor complexes, which propagate the signal by phosphorylating receptor regulated (R)-SMADs. TGF-β/activin type I receptors, also termed activin receptor-like kinase (ALK) 5 and 4, phosphorylate SMAD2 and SMAD3, whereas the corresponding BMP receptors phosphorylate SMAD1, 5, and 8. All these R-Smads, subsequently, can form complexes with the common-partner (co-)SMAD: SMAD4 [2,21] (Figure 2). 

Receptor activated SMAD complexes induce, amongst others, the expression of the third subclass of SMADs, the so called inhibitory SMAD (I-SMADS: SMAD6 and SMAD7). The I-Smads only share the MH2 domain with the other SMADs and can counteract the signals transduced by TGF-β-receptors as part of feedback loops; they act amongst others by competing with R‑SMADs for receptor binding, thereby inhibiting R-SMAD phosphorylation [22]. I-SMADs also can associate with the SMAD-ubiquitination-related factors (SMURF) 1 and SMURF 2, two HECT E3 ligases*,* to target the TGF-β-receptor complexes for degradation [23,24]. The stability of various SMAD proteins is also controlled by poly-ubiquitination. SMAD1 is poly-ubiquitinated by SMURF1/2 and carboxyl terminus of Hsc70-interacting protein (CHIP) [25,26,27]. SMAD2 is poly-ubiquitinated by SMURF2, NEDD4L and WWP1 [28,29,30]. SMAD3 is poly-ubiquitinated by CHIP [31], and SMAD7 has been shown to be targeted for poly-ubiquitination by ARKADIA and RNF12 [32,33,34].

## 2. Critical Regulation of SMADs by Mono-Ubiquitination 

Both in TGF-β superfamily induced SMAD and non-SMAD signaling the involvement of multiple E3 ligases and distinct types of polyubiquitination chains (*i*.*e*., degradation-directed lysine-48 chains and signaling-directed lysine 63-chains) has been solidly established. Mono-ubiquitination, however, was only relatively recently uncovered as an important regulatory mechanism for activation and inactivation of SMADs.

### 2.1. Mono-Ubiquitination of SMAD3 at Multiple Lysines 

**Figure 3:**
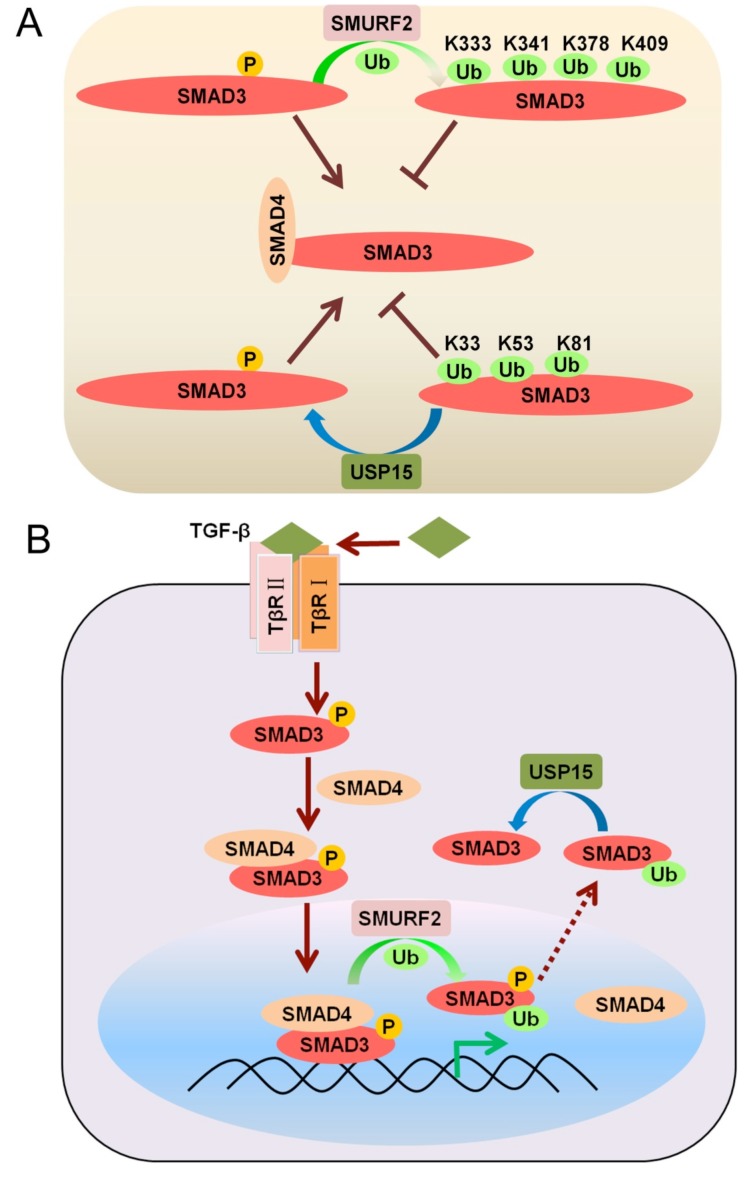
(**A**) SMURF2 and USP15 control the mono-ubiquitination state of SMAD3 (**B**) Mono-ubiquitination of SMAD3 by SMURF2 in the nucleus disrupts SMAD complexes.

In addition to targeting the TGF-β receptor for poly-ubiquitination via I-SMADs [23], the E3 ligase SMURF2 was recently found to also promote SMAD3 mono-ubiquitination (Figure 3A). Four different lysine residues (K333, K341, K378, and K409, located in the MH2 domain of SMAD3), were identified as SMURF2 target sites. SMURF2-induced multiple mono-ubiquitination of SMAD3 was found to inhibit formation of homotrimeric SMAD3 and heterotrimeric SMAD3-SMAD4 complexes, and, as a consequence, limit the binding of SMADs to DNA [35] (Figure 3A,B).

In another study SMAD3 activation and its nuclear entry were observed to correlate with its mono-ubiquitination state. Moreover, DNA binding assays showed that mono-ubiquitinated SMAD3 has less affinity for the SMAD binding element (SBE), suggesting that mono-ubiquitination of SMAD3 in the nucleus functions as an inhibitory modification for the transcriptional activity of SMAD3. Intriguingly, analysis of a series of SMAD3 mutants, bearing lysine-to-arginine substitutions in evolutionarily conserved residues, identified lysine 81, lysine 33, and lysine 53 as the major mono-ubiquitination sites in this study. Consistently, structural analysis showed that lysine 81 is a key residue in the highly conserved MH1 DNA-binding domain of the SMADs. In addition, this study identified USP15 as a deubiquitinating enzyme specific for SMAD3 mono-ubiquitination. USP15 can remove multiple mono-ubiquitin conjugations from SMAD3 and thus seems to be required for SMAD3 transcriptional activation [36] (Figure 3B). 

It remains to be established whether the mono-ubiquitination state of these distinct lysines depends on the cell type and cellular context, on the ubiquitination state of SMAD4 (see below), and/or on the activation state of specific signaling cascades. 

### 2.2. Mono-Ubiquitination of SMAD4

**Figure 4:**
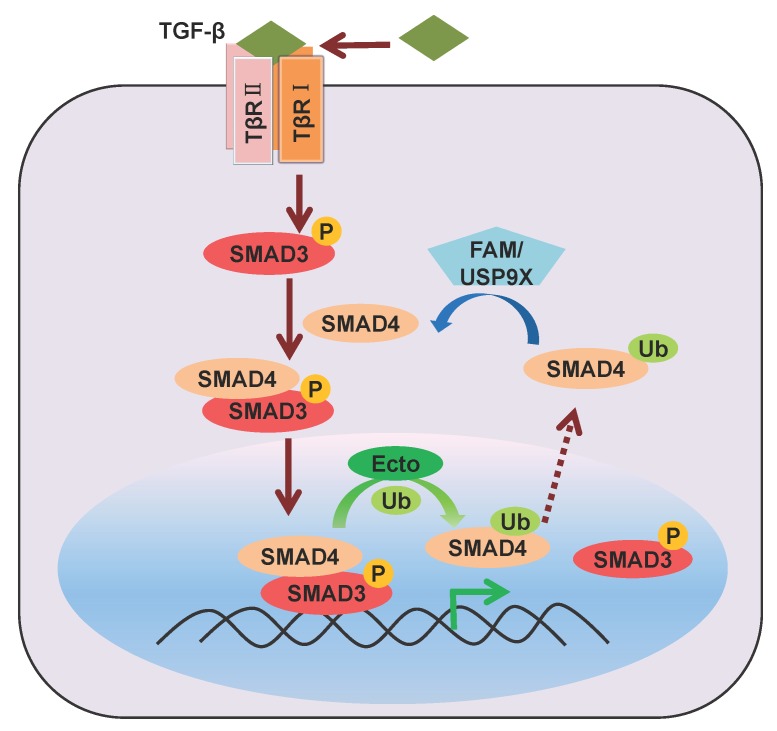
Regulation of SMAD4 mono-ubiquitination by Ectodermin and FAM/USP9X.

As a common-partner for the R-SMADs, SMAD4 is a central mediator of both TGF-β/activin and BMP signaling and, thus, a critical regulator of the biological effects of TGF-β family ligands. Unlike R-SMADs, SMAD4 has not been reported to be phosphorylated by TGF-β receptors. Although, under non-stimulated conditions, SMAD4 is located throughout the cytoplasm and nucleus, fusion of a single ubiquitin to SMAD4 was found to promote its accumulation in the cytoplasm, suggesting a possible role for mono-ubiquitination of SMAD4 [37]. Ectodermin (Ecto)/Tif1g/TRIM33, previously proposed to be a SMAD4 poly-ubiquitination ligase, was actually found to function as a SMAD4 mono-ubiquitination ligase in the nucleus [38,39]. Mono-ubiquitination on SMAD4 lysine 519 inhibits SMAD2-SMAD4 complex formation and disrupts the binding between phosphorylated SMAD2 and SMAD4 on the chromatin. Therefore, Ectodermin-mediated mono-ubiquitination of SMAD4 represents one of the mechanisms to inhibit TGF-β signaling (Figure 4). 

In line with the role of SMAD4 as common-partner, FAM/Usp9x was identified as an SMAD4 deubiquitinating enzyme (DUB) that is critical for both TGF-β and BMP responsiveness in human cells and Xenopus embryos. FAM/Usp9x deubiquitinates the monoubiquitinated SMAD4 (which is exported from the nucleus (Figure 4)) and thereby enables SMAD2-SMAD4 complex formation. Thus, FAM and Ecto reversely regulate SMAD4 function in different sub-cellular compartments [39]. This post-translational modification circuit of SMAD4 provides insight into the mechanisms by which activated SMAD complexes are removed from target genes. 

**Figure 5:**
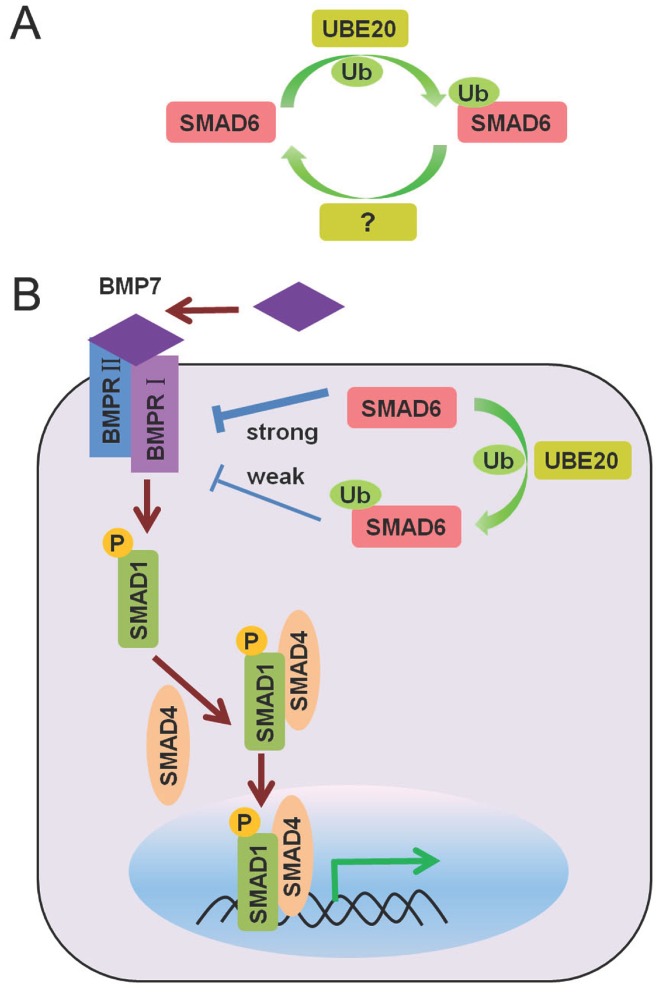
(**A**) Proposed model for regulation of SMAD6 mono-ubiquitination by UBE2O and a hypothetical DUB; (**B**) Mono-ubiquitination of SMAD6 impairs its binding to the BMP type I receptor thereby elevating BMP/SMAD signaling.

### 2.3. Mono-Ubiquitination of SMAD6

The I-SMAD SMAD6 is a crucial negative feedback regulator that can antagonize BMP/SMAD signaling via direct interaction with BMP type I receptors. Unlike the other SMADs, little is known regarding its post-translational modifications. Intriguingly, the putative E2 ubiquitin-conjugating enzyme UBE2O (E2-230K) was recently identified as a novel interacting protein of SMAD6 by mass spectrometry analysis. Lysine 174 of SMAD6 was subsequently observed to be mono-ubiquitinated by UBE2O. Cysteine 885 in the E2 active site of human UBE2O was found to be necessary for UBE2O to function as an E2-E3 hybrid enzyme for SMAD6 mono-ubiquitination [40] (Figure 5A). Mono-ubiquitinated SMAD6 was predominantly localized in the cytoplasm. More importantly, mono-ubiquitinated SMAD6 displayed reduced binding affinity for the activated BMP7 type I receptor (ALK2) (Figure 5B). Thus, through mono-ubiquitination of SMAD6 UBE2O can impair SMAD6 function and thereby trigger a higher level of BMP/SMAD signaling. In line with this, UBE2O was found to potentiate BMP7-induced adipocyte differentiation [40]. A DUB for mono-ubiquitinated SMAD6, thereby reversing the action of UBE20 (Figure 5A), still has to be identified. 

## 3. Conclusions and Future Perspectives

Ubiquitination has traditionally been viewed in the context of (lysine 48-linked) poly-ubiquitination, which targets substrate degradation via the proteasome. In contrast, mono-ubiquitin conjugation on a single residue or multiple sites mainly alters protein interactions and/or sub-cellular localization without an effect on protein stability. However, these distinct types of ubiquitination might in fact be inter-related. The tumor suppressor p53 is well known to be degraded and, thereby, maintained at low levels via poly-ubiquitination by Mdm2, an oncogenic E3 ligase. However, it has been reported that only higher levels of Mdm2 promote p53 poly-ubiquitination and nuclear degradation, whereas lower levels of Mdm2 in fact induce mono-ubiquitination and nuclear export of p53 [41]. Similar ubiquitination-mediated control has been described for the phosphoinositide 3-kinase/AKT cell survival pathway, which is antagonized by PTEN, a plasma-membrane lipid-phosphatase. PTEN is mono-ubiquitinated in the cytoplasm by NEDD4-1, which allows for PTEN nuclear import. The mono-ubiquitinated PTEN is stable in nuclei, while poly-ubiquitination of PTEN in the cytoplasm leads to its degradation [42]. Mono-ubiquitination also plays a key role in histone modifications. Mono-ubiquitination of histone H2B at lysine 120 (H2Bub1) by the RNF20-RNF40 E3 complex has been shown to induce a more open chromatin structure accessible to transcription factors and DNA repair proteins [43]. 

As a crucial signaling pathway for both development and cancer progression, the TGF-β/SMAD pathway, and its different levels of regulation, have been studied in great detail. While the core components including receptors and SMADs have been known to be poly-ubiquitinated for a relatively long time, the more recent studies focused on mono-ubiquitination of SMADs and its effects on signaling. Several reports have analyzed the (effects of) mono-ubiquitination of the Co-, R-, and I-SMADs SMAD3, SMAD4 and SMAD6 (Table S1). Mono-ubiquitination was found to alter R-SMAD and Co-SMAD complex formation, transcriptional complex assembly, or the interaction between the I-SMAD and the TGF-β receptor. Apparently, conjugation of one or multiple mono-ubiquitin molecules to a SMAD protein is likely to interfere with its association with binding partners and thereby to impair its function. Interestingly, mono-ubiquitination in either SMAD3 or SMAD4 appears to counteract formation of transcriptionally active R-SMAD-Co-SMAD complexes. Moreover, multiple mono-ubiquitinated lysines in SMAD3 seem to be involved, which may depend on the cell type and cellular context, specific signaling cascades, and the mono-ubiquitination state of SMAD4. In the case of SMAD6, mono-ubiquitination appears to block binding of SMAD6 to the BMP type I receptor ALK2, thereby preventing SMAD6-dependent inhibition of BMP signaling. SMAD mono-ubiquitination thus appears to represent a mechanism for both negative and positive fine-tuning of SMAD transcriptional complex formation, and the concomitant nuclear/cytoplasmic shuttling. It remains to be established whether there also exists a tight inter-relationship between mono-ubiquitination and poly-ubiquitination in the case of these TGF-β components.

As an important pathway for cancer initiation and progression, TGF-β/SMAD signaling needs to be tightly controlled to sustain homeostasis and to avoid detrimental responses. Uncontrolled activation of the TGF-β/SMAD system leads to unrestrained cellular responses and results in serious disorders in malignant tumors such as invasion and cancer metastasis. To avoid inappropriate over-activation, multiple negative regulating mechanisms are engaged to serve this purpose. SMAD3/SMAD4 mono-ubiquitination appears to represent one of these negative control mechanisms, and defects or absence of the corresponding E3 ligase or abnormal high expression of the involved DUB(s) could thus enhance aggressive cancer progression. 

A couple of DUBs have been identified that counteract mono-ubiquitination of SMADs. Multiple mono-ubiquitination of SMAD3 can be removed by USP15 and mono-ubiquitination of SMAD4 seems to be removed by FAM/USP9X. Mono-ubiquitination is thus a reversible process, which suggests that continuous cycles of SMAD mono-ubiquitination and deubiquitylation are required to properly regulate the levels of TGF-β signal transduction. In line with this, abnormal highly expressed USP15 in glioblastoma correlates with TGF-β/SMAD activation [44]. In Table S1, we summarized the known mono-ubiquitination sites in SMADs, their function, and the involved E3 ubiquitin ligases and DUBs. 

Another aspect that deserves discussion is the techniques used to identify mono-ubiquitination. Although mass spectrometry is a powerful technique to identify protein ubiquitination, it is still more accurate to confirm the mono-ubiquitinated site by making mutations in candidate lysine residues. By comparing the wild-type protein and its mutated version(s), one can distinguish mono-ubiquitination of a certain lysine residue from poly-ubiquitination, because mutation of a mono-ubiquitination site leads to the disappearance of single ubiquitinated band (usually migrating 8–15 KD higher than the non-ubiquitinated substrate), while mutation of a poly-ubiquitination site mitigates multiple bands or even the whole ubiquitinated ladder. One should then compare substrate ubiquitination with wild-type and lysine-free (K0) ubiquitination, because most substrates will be multi-ubiquitinated. Since a large amount of work might be needed to mutate all lysine residues in proteins, protein deletion mutants were usually analyzed first to minimize the mono-ubiquitinated region. This strategy was employed by multiple independent groups when searching for SMAD mono-ubiquitination [35,36,40]. 

In summary, multiple studies have identified reversible mono-ubiquitinaton and its regulatory potential for distinct SMAD signal transducers. These observations have substantially increased our knowledge on regulation of the activities of SMAD complexes and, thereby, of TGF-β superfamily signaling. Moreover, the established links between the ubiquitin modification system and the activity of SMADs provide new points of potential therapeutic intervention, in particular for diseases caused by unbalanced TGF-β activity.

## Supplementary Materials

Supplementary materials (Table S1) can be found at: http://www.mdpi.com/2073-4409/3/4/981/s1.
